# Diacylglycerol Kinases in the Coordination of Synaptic Plasticity

**DOI:** 10.3389/fcell.2016.00092

**Published:** 2016-08-31

**Authors:** Dongwon Lee, Eunjoon Kim, Keiko Tanaka-Yamamoto

**Affiliations:** ^1^Center for Synaptic Brain Dysfunctions, Institute for Basic ScienceDaejeon, South Korea; ^2^Department of Biological Sciences, Korea Advanced Institute of Science and TechnologyDaejeon, South Korea; ^3^Center for Functional Connectomics, Korea Institute of Science and TechnologySeoul, South Korea

**Keywords:** diacylglycerol kinase, synaptic plasticity, long-term potentiation, long-term depression, protein kinase C

## Abstract

Synaptic plasticity is activity-dependent modification of the efficacy of synaptic transmission. Although, detailed mechanisms underlying synaptic plasticity are diverse and vary at different types of synapses, diacylglycerol (DAG)-associated signaling has been considered as an important regulator of many forms of synaptic plasticity, including long-term potentiation (LTP) and long-term depression (LTD). Recent evidences indicate that DAG kinases (DGKs), which phosphorylate DAG to phosphatidic acid to terminate DAG signaling, are important regulators of LTP and LTD, as supported by the results from mice lacking specific DGK isoforms. This review will summarize these studies and discuss how specific DGK isoforms distinctly regulate different forms of synaptic plasticity at pre- and postsynaptic sites. In addition, we propose a general role of DGKs as coordinators of synaptic plasticity that make local synaptic environments more permissive for synaptic plasticity by regulating DAG concentration and interacting with other synaptic proteins.

## Introduction

Alterations in the efficacy of synaptic transmission are believed to be cellular mechanisms of learning and memory. Two well-studied forms of such synaptic plasticity are long-term potentiation (LTP) and long-term depression (LTD), which have been widely observed at different types of synapses in many brain areas (Citri and Malenka, 2008). The signaling mechanisms of synaptic plasticity vary to some extent depending on the types of synapses and stimulation. One of the main differences is the synaptic site of expression: some forms of LTP and LTD rely on changes in presynaptic neurotransmitter release, but others rely on changes in postsynaptic receptor numbers or properties (Malenka and Bear, 2004; Castillo, 2012; Huganir and Nicoll, 2013). Nevertheless, there are some properties shared by several forms of LTP and LTD. A common property of all forms of LTP and LTD is that potentiation and depression are triggered by transient stimulation, whereas the potentiation and depression last for a long time. For the long-term maintenance of altered synaptic strength, translational, and transcriptional regulations are often involved (Citri and Malenka, 2008). The protocol triggering LTP or LTD usually consists of strong or repeated synaptic stimulation, which likely activates synaptic receptors and triggers increases in the levels of signaling molecules, such as calcium and diacylglycerol (DAG). In fact, signaling mechanisms associated with calcium or DAG have been shown to be required for several forms of LTP and LTD (Sossin and Farah, 2009).

In many types of cells, including neurons, DAG is generally produced after the activation of G-protein coupled receptors (GPCRs) through the metabolization of phosphatidylinositol 4,5-bisphosphate by phospholipase C (PLC) (Rhee, 2001). At synapses, an increase in DAG level by the activation of GPCRs likely regulates its target molecules required for synaptic plasticity (Brose et al., 2004). A well-known target molecule of DAG is protein kinase C (PKC), which has been shown to be involved in many forms of LTP or LTD. Therefore, the regulation of DAG concentrations at synapses is crucial for the regulation of these forms of LTP or LTD.

The conversion of DAG to phosphatidic acid (PA) by diacylglycerol kinase (DGK) is the major pathway for the termination of DAG signaling (Sakane et al., 2007). Ten mammalian DGK isoforms have been identified so far, and at least 8 of them are readily detected in the mammalian brain, suggesting the important roles of DGKs in the brain (Tu-Sekine and Raben, 2011). Interestingly, the expression pattern of each isoform in the brain is different, and their subcellular localizations are distinct (Tu-Sekine and Raben, 2011; Ishisaka and Hara, 2014), suggesting that different isoforms of DGKs have unique neuronal or synaptic functions. In this review, we summarize the roles of DGKs in the regulation of synaptic plasticity (see Table 1 for summary), focusing on specific types of synaptic plasticity and individual DGK isoforms involved. In addition, we would like to emphasize the emerging notion that DGKs may be involved in the generation of local environments suitable for synaptic plasticity.

**Table 1:** **Involvement of DGK isoforms in several forms of synaptic plasticity**.

**DGK isoform**	**Synapse**	**Localization**	**Tested forms of synaptic plasticity**	**Reported effects in KO mice**	**Known/expected functions of DGKs**
DGKε	Perforant path-dentate granule cell synapses	N.D.	LTP	Reduction	Regulating amounts of DAG and PKC activity required for LTP, and regulating the lipid signaling leading to the production of retrograde messengers required for LTP
DGKζ	Hippocampal SC-CA1 synapses	Postsynaptic density	Postsynaptic LTP	Enhancement	Regulating amounts of DAG produced by mGluR activation, and balancing PKC activity, which is a modulator of LTP and LTD
			Postsynaptic LTD	Reduction	
DGKβ	Hippocampal SC-CA1 synapses	Membranes including synaptic areas	Postsynaptic LTP	Reduction	Regulating basal DAG levels
DGKι	Hippocampal SC-CA1 synapses	Presynaptic areas	Presynaptic LTD	Reduction	Reducing DAG levels and consequently preventing the activation of target molecules that antagonize LTD
DGKκ	Hippocampal SC-CA1 synapses	N.D.	Postsynaptic LTP	Reduction	Regulating basal DAG levels
			Postsynaptic LTD	Enhancement	
DGKζ	Cerebellar parallel fiber-Purkinje cell synapses	Postsynaptic areas	Postsynaptic LTP	Normal	–
			Postsynaptic LTD	Reduction	Targeting PKCα required for LTD at synapses, maintaining optimal PKCα activity levels via reducing basal DAG levels, and receiving inhibition from PKCα

N.D. stands for “not described or undetermined.”

## DGKs involved in several forms of hippocampal synaptic plasticity

### DGKε for LTP at perforant path-dentate granule cell synapses

The involvement of DGKs in synaptic plasticity was first demonstrated for LTP at synapses of hippocampal dentate granular cells receiving inputs from the perforant path of the entorhinal cortex. LTP at these synapses requires the action of postsynaptic NMDA-type glutamate receptors (NMDARs) and increases in calcium concentrations (Colino and Malenka, 1993; Kleschevnikov and Routtenberg, 2001). However, it is controversial whether this LTP is presynaptically or postsynaptically expressed. Several studies demonstrated that LTP at these synapses was expressed by an increase in the probability of presynaptic neurotransmitter release (Christie and Abraham, 1994; Wang et al., 1996; Min et al., 1998), which is controlled by the retrograde lipid messenger, platelet-activating factor (Kato and Zorumski, 1996; Chen et al., 2001). On the other hand, other studies showed that LTP was expressed by an increase in the number of postsynaptic AMPA-type glutamate receptors (Wang et al., 1996; Reid and Clements, 1999; Moga et al., 2006).

Despite the undefined locus of LTP expression, PKC has been demonstrated to be involved in this form of LTP. PKC activation rescues LTP blocked by an NMDAR antagonist (Kleschevnikov and Routtenberg, 2001), suggesting involvement of PKC activation in LTP. In contrast, PKC activation by metabotropic glutamate receptors (mGluRs) prior to LTP induction inhibits subsequent LTP induction (Gisabella et al., 2003), suggesting that maintaining minimum PKC activity at the basal state is required for LTP. These results indicate that the regulation of PKC activity is critical for LTP induction.

In mice lacking DGKε (DGKε^−/−^), which is the only DGK that can act on *sn*-2 arachidonoyl-DAG, LTP at dentate granular cell synapses was impaired (Rodriguez de Turco et al., 2001). A possible function of DGKε is to reduce DAG concentrations and PKC activity at the basal state, considering the dynamics of PKC activity required for LTP induction. Another possibility is that DGKε regulates lipid metabolism, so that the synthesis of platelet-activating factor is suppressed, leading to impairment of LTP in DGKε^−/−^ mice (Rodriguez de Turco et al., 2001).

### Role of DGKζ in postsynaptic LTP and LTD at SC-CA1 synapses

Synaptic plasticity has been extensively investigated at hippocampal Schaffer-collateral (SC)-CA1 synapses, in which LTP and LTD are postsynaptically expressed (Citri and Malenka, 2008). Many studies have demonstrated that PKC is involved in both LTP and LTD at SC-CA1 synapses (Akers et al., 1986; Malinow et al., 1989; Klann et al., 1993; Thiels et al., 2000), suggesting the importance of DAG regulation in the postsynaptic area. DGKζ is mainly present at postsynaptic sites and directly interacts with PSD-95 family proteins (Kim et al., 2009). In DGKζ^−/−^ mice, LTP at SC-CA1 synapses is enhanced, whereas LTD is reduced (Seo et al., 2012). Importantly, pharmacological inhibition of PLC and PKC restores abnormal LTP and LTD in DGKζ^−/−^ mice, suggesting that enhanced PLC-PKC signaling by DGKζ deficiency may lead to an altered balance of LTP and LTD (Seo et al., 2012). Therefore, DGKζ appears to limit excessive increases in DAG level and PKC activity for proper modulation of bidirectional synaptic plasticity at hippocampal SC-CA1 synapses.

### Role of DGKβ in postsynaptic LTP at SC-CA1 synapses

In addition to DGKζ, DGKβ has also been reported to regulate postsynaptically expressed LTP at hippocampal SC-CA1 synapses. However, its functions appear to be different from those of DGKζ, because LTP was reduced in DGKβ^−/−^ mice, contrary to the enhanced LTP in DGKζ^−/−^ mice (Shirai et al., 2010). DGKβ is expressed at high levels in the hippocampal pyramidal cell layer (Goto and Kondo, 1999) and shows unique localization patterns at the plasma membrane (Caricasole et al., 2002) and postsynaptic compartments (Hozumi et al., 2008). Thus, both DGKβ and DGKζ are localized around the postsynaptic area, excluding a possibility that this accounts for the differences in their functions. Another possibility is that DGKβ and DGKζ are responsible for metabolizing DAG under different contexts. DGKβ deficiency resulted in a reduction in PA production and an increase in DAG level even without stimulation (Shirai et al., 2010), whereas DGKζ deficiency resulted in a significant reduction in PA production only when stimulation was applied without changes under basal conditions (Kim et al., 2009). These results suggest that whereas DGKζ converts DAG to PA after synaptic stimulation to maintain DAG at appropriate levels that are required for synaptic plasticity, DGKβ is mainly responsible for lowering DAG levels at the basal state.

### Role of DGKι in mGluR-dependent, presynaptic LTD at SC-CA1 synapses

DGKι, which shares a similar domain structure with DGKζ, binds to PSD-95 (Yang et al., 2011). However, unlike DGKζ, DGKι is also present in axon terminals in addition to postsynaptic sites, being detected in the presynaptic plasma membrane and synaptic vesicles (Yang et al., 2011). In DGKι^−/−^ mice, postsynaptic LTP and LTD are not altered at hippocampal SC-CA1 synapses, presumably because DGKβ and DGKζ even in the absence of DGKι are functionally sufficient to regulate the postsynaptic DAG metabolism that is required for synaptic plasticity. In contrast, mGluR-dependent LTD at these synapses is suppressed in the hippocampus of neonatal (2-week old) DGKι^−/−^ mice. It has been shown that mGluR-dependent LTD in SC-CA1 synapses of neonatal mice relies mainly on the reduction of presynaptic release probability (Fitzjohn et al., 2001; Zakharenko et al., 2002; Rammes et al., 2003; Nosyreva and Huber, 2005; Yang et al., 2011). Consistently with the idea that presynaptic LTD is suppressed at DGKι^−/−^ SC-CA1 synapses, mGluR stimulation did not cause an activity-dependent reduction in release probability in these mice (Yang et al., 2011). Furthermore, inhibition of the binding of DAG to its target molecules or inhibition of PKC in DGKι^−/−^ mice rescued mGluR-dependent LTD as well as activity-dependent reduction of release probability (Yang et al., 2011). Thus, DGKι may work during normal mGluR-LTD to remove DAG at presynaptic terminals and to suppress the activity of DAG targets, such as PKC and Munc13, as well as enhancement of neurotransmitter release.

### Role of DGKκ in postsynaptic LTP and LTD at SC-CA1 synapses

A recent study demonstrated that the reduction of DGKκ expression levels using an shRNA resulted in reduced LTP and increased LTD at hippocampal SC-CA1 synapses (Tabet et al., 2016). This result is similar to the abovementioned reduced LTP observed in DGKβ^−/−^ mice, and suggests that DGKκ may also be required for lowering DAG levels under basal conditions and inducing normal LTP. In line with this idea, in knockout mice lacking the fragile X mental retardation protein where DGKκ translation is impaired, DAG levels are increased under basal conditions, but not after mGluR stimulation. It is possible that DGKκ may cooperate with DGKβ to maintain low levels of DAG under basal conditions for normal induction of LTP at SC-CA1 synapses.

## DGKζ is required for cerebellar LTD

In addition to hippocampal synaptic plasticity, DGKζ regulates cerebellar LTD that is postsynaptically expressed at the synapses of cerebellar Purkinje cells receiving inputs from parallel fibers, as supported by impaired LTD, but not LTP, in DGKζ^−/−^ mice (Lee et al., 2015). Cerebellar LTD has long been studied, and the importance of PKC in LTD has also been well established (Linden and Connor, 1991; De Zeeuw et al., 1998), with the PKC isoform PKCα being critical (Leitges et al., 2004). Our results showed that DGKζ bound to not only PSD-93, a PSD-95 relative abundant in Purkinje cells, but also to PKCα in Purkinje cells, and such binding functions of DGKζ were required for LTD (Lee et al., 2015). In addition, we have shown that LTD induction causes the dissociation of DGKζ and PKCα, and that the catalytic function of DGKζ is also required for LTD. These results collectively suggest the following mechanisms. PSD-93-bound DGKζ interacts with and promotes the synaptic localization of PKCα, but suppresses PKCα activity under basal conditions by reducing DAG concentrations. When LTD is triggered, PKCα dissociates from DGKζ and gets activated to promote the induction of cerebellar LTD.

## General roles of DGKs in synaptic plasticity

As summarized above, 5 different isoforms of mammalian DGKs have so far been reported to be involved in some forms of synaptic plasticity (Table 1). Generally, the 10 known isoforms of DGKs are categorized into five types based on their distinct functional domain structures, which display differential distribution patterns in the brain (Ishisaka and Hara, 2014). These diversities in the domain structures and distribution patterns of brain DGKs may influence their distinct subcellular localization, spectrum of binding proteins, and regulation of specific aspects of LTP and LTD.

**Figure 1:**
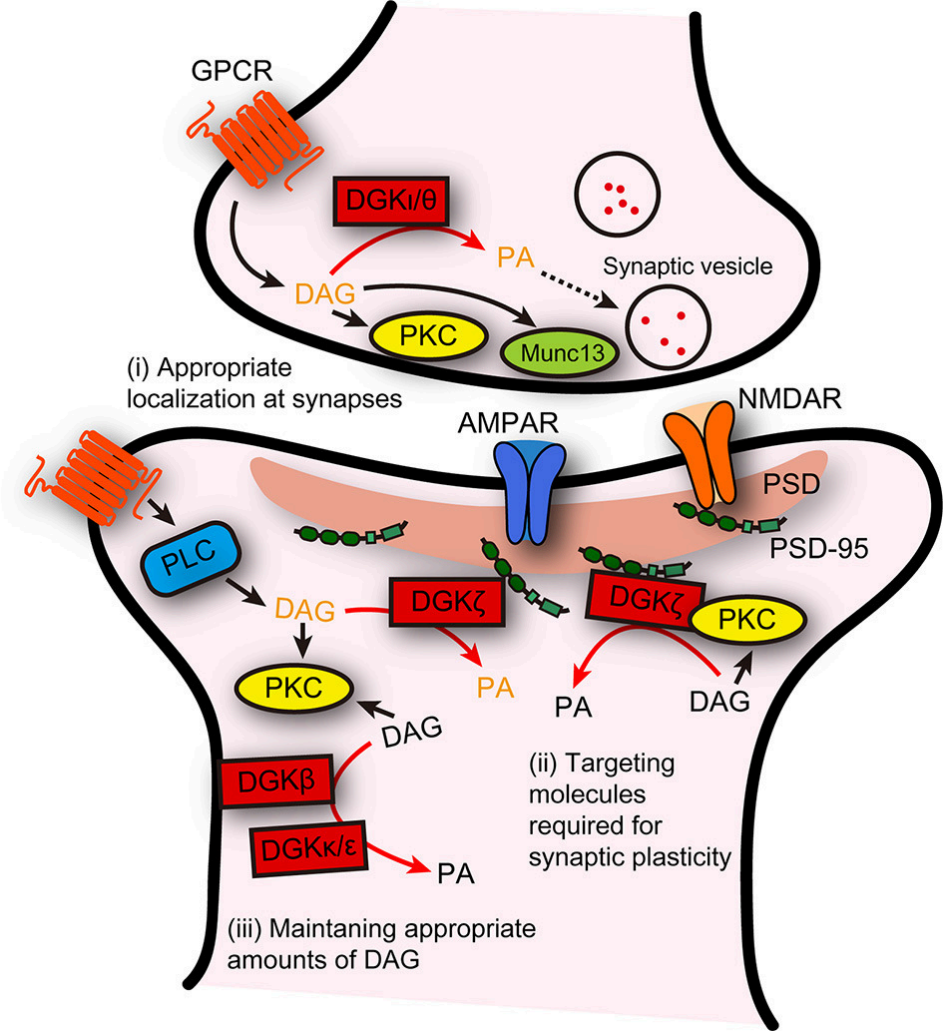
**Possible distinct functions of DGK isoforms, and their general role as a coordinator of synaptic plasticity**. Distinct functions of individual DGK isoforms in several forms of synaptic plasticity are summarized. In addition, DGKs could be considered to play a general role: DGKs act to create a synaptic environment that is suitable for triggering synaptic plasticity, through their abilities to (i) localize itself appropriately at presynaptic or postsynaptic areas, (ii) interact with and facilitate the synaptic targeting of the molecules required for triggering synaptic plasticity, and (iii) maintain DAG at levels that can adequately contribute to the induction of synaptic plasticity. DAG/PA in black and in dark yellow indicates DAG/PA produced under basal conditions and after stimulation, respectively.

Although, the current results suggest that individual DGK isoforms distinctly regulate several forms of LTP or LTD, they can be considered to play a conceptually common role in synaptic plasticity. In general, molecules involved in synaptic plasticity fall into two categories based on their functions, namely, mediators or modulators. Mediators are directly responsible for triggering synaptic plasticity, whereas modulators are molecules modulating the ability to trigger synaptic plasticity or playing a permissive role (Citri and Malenka, 2008). Given that all isoforms of DGKs described above do not directly mediate the expression of synaptic plasticity, they should be categorized as modulators. Furthermore, DGKs may be specifically considered as coordinators because they function to prepare synapses to undergo synaptic plasticity. As described above, in some cases, DGKs reduce DAG signaling under basal conditions so that DAG signaling can be effectively enhanced after the induction of synaptic plasticity. In other cases, DGKs control DAG signaling “after” the initiation of the induction of synaptic plasticity. Therefore, DGK-dependent modulation of DAG tones before and after the induction of synaptic plasticity may create synaptic environments appropriate for triggering synaptic plasticity. Such a coordinating role would be accomplished by the ability of DGKs to localize itself at synapses, to bind, recruit, and temporally suppress the activity of downstream effector molecules of DAG, such as PKCα, required for synaptic plasticity, and to timely terminate DAG signaling to suppress excessive induction of synaptic plasticity (Figure 1).

## Remaining questions

Studies using knockout mice of specific isoforms of DGKs have demonstrated the involvement of DGKs in some forms of synaptic plasticity, and based on these studies, the general role of DGKs in synaptic plasticity has been proposed. The next question that naturally arises is whether other DGK isoforms that are abundantly expressed in the brain, such as DGKα, DGKγ, DGKη, and DGKθ, also play roles in some forms of synaptic plasticity. Future studies to address this question should support or even strengthen the idea that DGKs function as coordinators of synaptic plasticity.

Although, we have described some molecular mechanisms as to how DGK isoforms are involved in synaptic plasticity, there are still several questions regarding the molecular mechanisms, including the two following straightforward ones. The first is why there are three isoforms of DGKs–DGKβ, DGKζ, and DGKκ–that are required for the regulation of synaptic plasticity in hippocampal CA1 synapses. One possibility is that they play distinct roles based on their specific subcellular localizations and catalytic properties: DGKζ localized at synapses via binding with PSD-95 (Kim et al., 2009) may metabolize only high concentrations of DAG produced after synaptic stimulation, while DGKβ and DGKκ may be capable of metabolizing low concentrations of DAG around synapses (Figure 1). Although, the subcellular localization of DGKκ in neurons still remains unclear, the reported specific localization of DGKβ at the plasma membrane (Caricasole et al., 2002) may render distinct functions to DGKκ and DGKβ under basal conditions.

The second question is how DGK isoforms are localized to presynaptic terminals to regulate presynaptically expressed synaptic plasticity. DGKι is shown to be involved in presynaptic LTD (Yang et al., 2011). Although, the involvement of DGKθ in synaptic plasticity is not directly tested, DGKθ was shown to regulate synaptic vesicle recycling presumably via the production of PA (Goldschmidt et al., 2016). Except for common structural domains of DGKs (C1 and catalytic domains) DGKι and DGKθ do not share domains that may mediate the interaction with presynaptic molecules. Identification of such mechanisms would further advance our understanding of the DGK-dependent regulation of presynaptically expressed synaptic plasticity.

Considering that DGKs create synaptic environments appropriate for triggering synaptic plasticity, DGKs may be adequate regulators of metaplasticity. Metaplasticity refers to activity-dependent synaptic changes that modulate the ability to induce subsequent synaptic plasticity (Abraham and Bear, 1996). The activities of DGKs likely rely on their localization or posttranslational modifications (Shulga et al., 2011), and DGK protein levels may also alter the overall activity of DAG metabolism. Therefore, it is possible that activity-dependent regulation of DGK protein levels, localization, or modification leads to metaplasticity. Intriguingly, it has recently been shown that the cellular microRNA miR-34a targets DGKζ mRNA, and DGKζ expression was decreased via the stimulation-dependent upregulation of miR-34a in immune cells (Shin et al., 2013). A similar regulation of DGKζ or other DGK isoforms may be achieved at synapses as mechanisms of metaplasticity.

Finally, it has not yet been intensively investigated as to how DGK-dependent regulation of synaptic plasticity contributes to learning and memory, although a study has shown that DGKβ is necessary for hippocampus-dependent spatial reference memory formation (Shirai et al., 2010), for which LTP at CA1 synapses is implicated (Martin et al., 2000). To avoid compensation by other DGKs or other mechanisms in knockout mice, it would be needed to employee additional approaches such as conditional knockout of DGKs, mutations in specific domains, or temporal control of such modifications. Nevertheless, considering the role of DGKs in the coordination of synaptic environments for synaptic plasticity, it would be highly valuable to understand how DGK-dependent regulations of synaptic plasticity affect learning and memory at the behavior level.

## Author contributions

All authors listed, have made substantial, direct and intellectual contribution to the work, and approved it for publication.

## Funding

This work was supported by the Korea Institute of Science and Technology Institutional Program (Project No. 2E26190) and the Institute for Basic Science (IBS-R002-D1).

### Conflict of interest statement

The authors declare that the research was conducted in the absence of any commercial or financial relationships that could be construed as a potential conflict of interest.
